# Improving breastfeeding support through the implementation of the baby friendly hospital and community initiatives: a scoping review protocol

**DOI:** 10.12688/hrbopenres.13180.2

**Published:** 2021-04-23

**Authors:** Aisling Walsh, Pieternella Pieterse, Zoe McCormack, Ellen Chirwa, Anne Matthews

**Affiliations:** 1 Department of Public Health and Epidemiology, Royal College of Surgeons in Ireland, Dublin, Ireland; 2 School of Nursing, Psychotherapy & Community Health, Dublin City University, Dublin, Ireland; 3 Kamuzu College of Nursing, University of Malawi, Blantyre, Malawi

**Keywords:** Breastfeeding, Baby-friendly hospital initiative, Baby-friendly community initiative, scoping review.

## Abstract

**Background: **Improved breastfeeding practices have the potential to save the lives of over 823,000 children under 5 years old globally every year. Exclusively breastfeeding infants for the first six months would lead to the largest infant mortality reduction. The Baby-Friendly Hospital Initiative (BFHI) is a global campaign by the World Health Organization and the United Nations Children's Fund (UNICEF), which promotes best practice to

support breastfeeding in maternity services. The Baby-Friendly Community Initiative (BFCI) is an extension of the BHFI’s 10 ^th^ step of the Ten Steps to Successful Breastfeeding and of the BFHI overall. Its focus is on community-based breastfeeding supports for women. There have been no known attempts to synthesise the overall body of evidence on the BFHI in recent years, and no synthesis of empirical research on the BFCI. This scoping review asks the question: what is known about the implementation of the BFHI and the BFCI globally?

**Methods and analysis: **This scoping review will be conducted according to the Joanna Briggs Institute methodology for scoping reviews. Inclusion criteria will follow the Population, Concepts, Contexts approach. A data charting form will be developed and applied to all the included articles. Qualitative and quantitative descriptive analysis will be undertaken. The PAGER (Patterns, Advances, Gaps, Evidence for practice and Research recommendations) methodological framework will be used to analyse and report review findings.

**Conclusion:** This review will establish gaps in current evidence which will inform areas for future research in relation to this global initiative.

## Introduction

Globally, improved breastfeeding practices have the potential to save the lives of over 823,000 children under 5 years old every year, and save $300US billion ^1^. Exclusively breastfeeding infants for the first six months of their life is known to be the best start for a baby and a more widespread adoption of exclusive breastfeeding would lead to the largest infant mortality reduction ^1^. It can contribute towards meeting Sustainable Development Goals (SDG) 2 and 3 - targets on nutrition and health - as well as being linked to many other SDGs. Since 1990, the World Health Organization (WHO) recommends that all newborn babies are exclusively breastfed for the first six months of their lives, and continue to be breastfed for up to two years. Currently, just 42.2% of infants under 6 months are being exclusively breastfed and just 33 countries are on target for exclusive breastfeeding ^2^. Breastfeeding rates are both supported and hindered by the social determinants of health and multi-level support is needed, including policy, health systems and services, communities and families ^3^. ****

The Baby-Friendly Hospital Initiative (BFHI), launched by WHO and United Nations Children's Fund (UNICEF) in 1991, has been implemented globally in over 150 countries and is a pillar of the WHO/UNICEF Global Strategy for Infant and Young Child Feeding ^4^. One of the nine operational targets of the *Global Strategy for Infant and Young Child Feeding* is to ensure that every maternity facility practices the Ten Steps to Successful Breastfeeding. Hospitals or maternity facilities that comply with the Ten Steps to Successful Breastfeeding and with the International Code of Marketing of Breastmilk Substitutes and subsequent relevant World Health Assembly resolutions (the Code) are designated as “Baby-friendly”. Table 1 details the Ten Steps, which were updated and revised in 2018 ^5^. Although the BFHI has been widely implemented, coverage at a global level remains low. In 2017, just 10% of infants in the world were born in a facility currently designated as “Baby-friendly” ^5^. In 2018, the BFHI was revised, which led to greater emphasis on scaling up to universal coverage, ensuring sustainability, and integrating the programme more fully with health-care systems ^5^.

**Table 1.** Ten Steps to Successful Breastfeeding ^5^.

**Critical management procedures:**	
**1a**	Comply fully with the *International Code of Marketing of Breast-milk Substitutes* and relevant World Health Assembly resolutions
**1b**	Have a written infant feeding policy that is routinely communicated to staff and parents
**1c**	Establish ongoing monitoring and data-management systems
**2**	Ensure that staff have sufficient knowledge, competence and skills to support breastfeeding
**Key clinical practices**	
**3**	Discuss the importance and management of breastfeeding with pregnant women and their families
**4**	Facilitate immediate and uninterrupted skin-to-skin contact and support mothers to initiate breastfeeding as soon as possible after birth
**5**	Support mothers to initiate and maintain breastfeeding and manage common difficulties.
**6**	Do not provide breastfed newborns any food or fluids other than breast milk, unless medically indicated
**7**	Enable mothers and their infants to remain together and to practice rooming-in 24 hours a day
**8**	Support mothers to recognise and respond to their infants’ cues for feeding
**9**	Counsel mothers on the use and risks of feeding bottles, teats and pacifiers
**10**	Coordinate discharge so that parents and their infants have timely access to ongoing support and care

The Baby-Friendly Community Initiative (BFCI) is an extension of the BHFI’s 10 ^th^ step of the Ten Steps to Successful Breastfeeding and of the BFHI overall ^6^. Its focus is on community-based breastfeeding supports for women. Given the usual short post-partum stay in facilities, this 10 ^th^ step and associated separate initiatives are often critical to support breastfeeding mothers beyond the initial days of giving birth. The 10 ^th^ BFHI step changed from “foster the establishment of support groups and refer mothers to them on discharge from hospital” in the 1989 version to “coordinate discharge so that parents and their infants have timely access to ongoing support and care” in the revised version in 2018 ^7^. It has been suggested that this change in step 10 signals a shift in increased responsibility of facilities in planning and facilitating community supports for mothers ^7^. While the BFHI was adopted in 152 countries, it appears that the BFCI has been adopted in a smaller number of countries, namely low- and middle-income countries (LMICs), including Kenya, Cambodia, Gambia ^6^ and High Income Countries (HICs) such as Italy ^8^ and the UK ^9^ . 

Between 2012 and 2017, almost 80% of live births occurred with the assistance of skilled health personnel globally ^10^. However, the estimated coverage of births attended by skilled health personnel during this period shows significant inequality between WHO regions. Just 59% of the births in the sub-Saharan Africa Region (during the period 2012–2017) were attended by skilled health personal, where maternal mortality is highest ^10^. In other WHO regions, between 68% to 99% of all births were attended by skilled health personnel ^10^. This underlines the importance of supports at the community level for breastfeeding, and it points towards the need for international interventions that promote breastfeeding to be mindful of the need to improve equity of access to breastfeeding supports.

### Study rationale, aims and objectives

Scoping reviews are useful when a body of literature has not yet been comprehensively reviewed, or *‘exhibits a large, complex or heterogeneous nature, not amenable to a more precise systematic review’* ^11^. Scoping reviews map the range of evidence, and also identify gaps in the knowledge base, clarify concepts, and document research that inform and address practice ^12^. A pilot search as part of the initial stage of this review (see *Extended data*) found that the majority of articles in this area have been published since 2012. There have been no known attempts to synthesise the overall body of evidence on the BFHI in recent years, and no synthesis of empirical research on the BFCI.

## Methods and analysis

This scoping review will be conducted according to the Joanna Briggs Institute (JBI) methodology for scoping reviews ^13^. We will use the framework for scoping reviews developed by Arksey & O’Malley ^14^ as the foundation, updated and advanced by Levac *et al.* ^15^ and progressed further by new guidance from the JBI ^11, 13, 16^.

According to this framework, there are six different stages, including: 1) identifying the research question; 2) identifying relevant articles; 3) study selection; 4) charting the data; 5) collating, summarising and reporting results; and 6) consulting with stakeholders. The scoping review will also adhere to the Preferred Reporting Items for Systematic Reviews and Meta-Analyses Extension for Scoping Reviews (PRISMA-ScR) to ensure rigour in reporting. The review is registered with the Open Science Framework, DOI: https://doi.org/10.17605/OSF.IO/27R3M.

### Stage 1: identifying the research question

The research aims and objectives for this scoping review were developed iteratively through discussions between the research team and were informed by the pilot search of the literature. The proposed review is situated within a wider research project, which is evaluating the implementation of evidence-based policy on infant feeding in Malawi ^17^, focused on exclusive breastfeeding for the first six months. In Malawi, the BFHI has been a well-known vehicle for the improvement of exclusive breastfeeding promotion in hospitals and health facilities since 1993, and as recent as 2018, several externally funded initiatives have been implemented to revive the BFHI, provide training for healthcare staff and accreditation for baby-friendly hospitals ^6^.

A broad scoping exercise was undertaken by our research team in 2019 to examine empirical studies that have focused on the implementation of the BFHI in Africa ^18^. During the literature search the following topics were examined: healthcare professionals’ knowledge and attitudes towards the BFHI ^19– 21^ compliance with the BFHI code ^22^ and the implementation of the BFCI ^6, 7, 23, 24^. At this time, we have decided to focus on conducting a more systematic scoping review that incorporates both LMICs and HICs in order to provide up to date evidence and to identify knowledge gaps.

To our knowledge there have been two attempts to systematically synthesise the evidence on BFHI. Semenic and colleagues ^25^ undertook an integrative review synthesising barriers and facilitators to implementing the BFHI. A systematic review by Perez-Escamilla and colleagues ^26^ focused on the impact of the BFHI on child health outcomes up to 2012. This review concluded that adherence to the 10 Steps impacts early initiation of breastfeeding, exclusive breastfeeding and total duration of breastfeeding. In addition, UNICEF has documented case studies of the experiences of 13 countries in implementing the BFHI, across high, middle and low/incomes countries ^27^.

This scoping review asks the question: what is known about the implementation of the BFHI and the BFCI globally? The aim is to map and examine the evidence relating to the implementation of BFHI and BFCI globally. Review objectives include:

1. To provide an overview of interventions and/or approaches to implement the BFHI/BFCI
2. To identify barriers and enablers to implementation of the BFHI/BFCI
3. To identify knowledge gaps in relation to research on the BFHI/BFCI

### Stage 2: identifying relevant articles

***Search strategy.*** A three step search strategy, as documented in the JBI manual ^16^ will be followed. Step one is a limited search for peer-reviewed, published papers on the PubMed and CINAHL databases (see *Extended data* ^28^), which has already been performed. An academic research librarian was consulted and an analysis of the words contained in the titles, abstracts and index terms generated the list of keywords detailed in *Extended data*. Search terms will be piloted to assess the appropriateness of databases and keywords. The second step will be conducted with the librarian which may involve refining the search terms. The third step will be examining the references of key articles that have been identified for full text review that meet the inclusion criteria. The following databases have been selected in consultation with an academic librarian: Pubmed, Embase, Web of Science, Global Health and CINAHL. Key words and index terms from the title and abstract of key articles were noted and used to inform the search strategy. The timeframe for the search will be from when the first article was published in a given database to October 2020.

***Inclusion and exclusion criteria.*** Inclusion criteria will be guided by the Population, Concepts, Contexts approach ( Table 2) ^15^.

**Table 2.** Population, Concepts, Contexts.

Criteria	Determinants
Population	Women are pregnant, postnatal period and up to 2 years post-partum
Concepts	Baby-Friendly Hospital Initiative or the Baby-Friendly Community Initiative
Context	Hospital or community. No country or geographic location will be excluded.

All research designs will be included: qualitative, quantitative and mixed method studies. Quantitative studies will include both experimental (e.g., randomised trials, non-randomised trials) and observational (e.g., cohort, cross-sectional) study designs. Qualitative studies will include designs such as grounded theory, ethnography, phenomenology, action research and qualitative descriptive design. In addition, all types of reviews of empirical research will be included. Grey literature will not be considered for inclusion in the review.

Draft inclusion and exclusion criteria will be tested on a sample of 15 articles to check the criteria’s suitability and will be amended as necessary.

*Inclusion criteria*

Studies that:

i) describe the implementation of the BFHI and/or BFCI
ii) evaluate the BFHI (any of the 10 steps) and/or the BFCI
iii) focus on experiences of accessing/delivering supports and services through any of the ten steps of the BFHI and/or BFCI
iv) focus on breastfeeding outcomes as a result of the BFHI and/or BFCI
v) focus on any country or group of countries
vi) are in the peer reviewed literature only
vii) empirical studies and literature reviews of empirical research

*Exclusion criteria*

Studies that:

i) focus on other breastfeeding initiatives, supports/interventions in the hospital and/or community other than the BFHI/BFCI
ii) are published in a language other than English
iii) commentaries, conference proceedings, opinion pieces, letters, editorials, trial registrations, evaluation reports, abstracts, theses and book chapters

### Stage 3: study selection

The screening process will consist of two phases: i) a title and abstract screening; ii) full-text screening. In stage i) all titles and abstracts will be screened by two reviewers, one reviewer will review 100% of articles, and two other reviewers will review 50% each. Screening will be undertaken through Covidence and duplicates will be removed. Where there is disagreement between reviewers as to whether an article should be included or excluded, a third reviewer will arbitrate. At full text screening stage, one reviewer will undertake a full text screening for eligibility and 100% of articles will be cross-checked by another reviewer.

### Stage 4: charting the data

Data will be extracted according to the JBI framework ^13^. A data charting form will be developed and applied to all the included articles. Examples of information to be included in the data charting form is included in *Extended data* ^28^. Two reviewers will independently pilot the form on a random sample of approximately five included articles. Data will be coded and entered in Microsoft Excel. In keeping with scoping review methodology, an assessment of the quality of individual articles will not be undertaken. As the quality of the evidence will not be assessed, there are limits to the extent to which conclusions can be drawn about the evidence gap.

### Stage 5: collating, summarising and reporting results

A ‘descriptive-analytical’ method will be used. As this is a scoping review, it is not anticipated that aggregation and synthesis of individual research results will be undertaken. The PAGER (Patterns, Advances, Gaps, Evidence for practice and Research recommendations) methodological framework ^29^ will be used to analyse and report review findings. This will firstly consist of a descriptive summary of the included findings. A patterning chart will be developed, which will then allow for identification of advances in the field of research on the BFHI/BFCI. Gaps will then be identified from this analytical process. A framework for reporting evidence for practice will include consideration of the following stakeholders: policy makers; research commissioners and practitioners/service providers. The research recommendations will build on the identification of gaps. These final two stages of the framework will be conducted in consultation with stakeholders at Stage 6 of the review. Key reflective questions at each stage of the framework will guide the analysis ^29^. 

### Stage 6: consulting with stakeholders

Findings from the review will be prepared for stakeholders who have expertise in relation to the BFHI and the BFCI. These will include researchers, practitioners and policy makers at the global level and at WHO regional levels.

### Research checklist

This scoping review protocol was drafted using the PRISMA-SCR extension checklist ^28, 30^.

## Study status

This study has completed the scoping stage, including piloting and refining search terms. Full database searches are ready to run and de-duplication and screening will begin in December 2020.

## Conclusion

The aim of this scoping review is to map and examine the evidence relating to the implementation of Baby-Friendly Hospital and Community Initiatives globally. Results will be published in a peer-reviewed journal and disseminated through conferences and/or seminars. This review will establish gaps in current evidence which will inform areas for future research in relation to this global initiative.

## Data availability

### Underlying data

No data is associated with this article.

### Extended data

Open Science Framework: Improving breastfeeding support through the implementation of the Baby Friendly Hospital Initiative and the Baby Friendly Community Initiative. https://doi.org/10.17605/OSF.IO/27R3M; registered at https://osf.io/g8zbq ^28^.

This project contains the following extended data:

- Search strategy pilot.
- Draft data charting form (Peters *et al.*, 2020).
- PRISMA-SCR extension checklist.

Data are available under the terms of the Creative Commons Zero "No rights reserved" data waiver (CC0 1.0 Public domain dedication).
