# Supplementary material for: Improving breastfeeding support through the implementation of the baby friendly hospital and community initiatives: a scoping review protocol
Source: HRB Open Res. 2021 Apr 23;4:1. Originally published 2021 Jan 4. [Version 2] doi: 10.12688/hrbopenres.13180.2 (PMC8142597; doi:10.12688/hrbopenres.13180.2)
Supplement: Peer review history

# Peer review: Reviewer response for version 2

**DOI:** 10.21956/hrbopenres.14454.r29310

**Published:** 2021-05-21

**Authors:** Linda Alinane Nyondo-Mipando

**Affiliations:** 1 School of Public Health and Family Medicine, Department of Health Systems and Policy, College of Medicine, University of Malawi, Blantyre, Malawi

I have reviewed the responses and changes made and I fine with them and so I approve this version.

Is the study design appropriate for the research question?

Yes

Is the rationale for, and objectives of, the study clearly described?

Yes

Are sufficient details of the methods provided to allow replication by others?

Yes

Are the datasets clearly presented in a useable and accessible format?

Partly

Reviewer Expertise:

NA

I confirm that I have read this submission and believe that I have an appropriate level of expertise to confirm that it is of an acceptable scientific standard.

# Peer review: Reviewer response for version 2

**DOI:** 10.21956/hrbopenres.14454.r29309

**Published:** 2021-05-13

**Authors:** Patricia Leahy-Warren, Helen Mulcahy

**Affiliations:** 1 School of Nursing and Midwifery, National University of Ireland, Cork, Ireland; 2 School of Nursing and Midwifery, University College Cork, Cork, Ireland; 3 National University of Ireland, Cork, Ireland

Thank you for this revised article, we have no further comments to make. Well done on enhancing your publication and we look forward to reading the full Scoping Review.

Is the study design appropriate for the research question?

Yes

Is the rationale for, and objectives of, the study clearly described?

Yes

Are sufficient details of the methods provided to allow replication by others?

Partly

Are the datasets clearly presented in a useable and accessible format?

Not applicable

Reviewer Expertise:

Patricia Leahy-Warren areas of research expertise include but not limited to: breastfeeding; perinatal mental health; transition to parenthood and social support. Helen Mulcahy areas of research include but not limited to: breastfeeding support; child health; and parental concern.

We confirm that we have read this submission and believe that we have an appropriate level of expertise to confirm that it is of an acceptable scientific standard.

# Peer review: Reviewer response for version 1

**DOI:** 10.21956/hrbopenres.14308.r28576

**Published:** 2021-03-09

**Authors:** Linda Alinane Nyondo-Mipando

**Affiliations:** 1 School of Public Health and Family Medicine, Department of Health Systems and Policy, College of Medicine, University of Malawi, Blantyre, Malawi

The proposal is relevant and well outlined. The authors should specify how the disagreements will be settled during the time of the review. This needs to be specified ahead of time.

 There seems to be a leaning towards Malawi as such the authors have to be specific if this review is Malawi specific or will include other countries as well. Either way, the direction as far as the setting is concerned and boundaries for the papers that will be included should be explicit. If it covers research beyond Malawi, then other works from other countries should be cited as well and Malawi should not stand out.

Is the study design appropriate for the research question?

Yes

Is the rationale for, and objectives of, the study clearly described?

Yes

Are sufficient details of the methods provided to allow replication by others?

Yes

Are the datasets clearly presented in a useable and accessible format?

Partly

Reviewer Expertise:

Health Systems and Policy, Maternal Newborn and Adolescent Health and HIV and AIDS researcher

I confirm that I have read this submission and believe that I have an appropriate level of expertise to confirm that it is of an acceptable scientific standard, however I have significant reservations, as outlined above.

# Response

**Published:** 2021-04-06

**Authors:** Aisling Walsh

**Affiliations:** Royal College of Surgeons in Ireland, Ireland

**The proposal is relevant and well outlined. The authors should specify how the disagreements will be settled during the time of the review. This needs to be specified ahead of time.**

 This is addressed in Stage 3: study selection. 

**There seems to be a leaning towards Malawi as such the authors have to be specific if this review is Malawi specific or will include other countries as well. Either way, the direction as far as the setting is concerned and boundaries for the papers that will be included should be explicit. If it covers research beyond Malawi, then other works from other countries should be cited as well and Malawi should not stand out.**

 This is a global scoping review. The review question asks:  what is known about the implementation of the BFHI and the BFCI globally?   Also see Table 2 context; also inclusion criteria v. The focus on Malawi in Stage 1 has largely been removed.

- **Are the datasets clearly presented in a useable and accessible format?**

**Partly**

 No datasets have been included in the protocol.

# Peer review: Reviewer response for version 1

**DOI:** 10.21956/hrbopenres.14308.r28641

**Published:** 2021-02-19

**Authors:** Patricia Leahy-Warren, Helen Mulcahy

**Affiliations:** 1 School of Nursing and Midwifery, University College Cork, Cork, Ireland; 2 National University of Ireland, Cork, Ireland; 3 School of Nursing and Midwifery, National University of Ireland, Cork, Ireland

Thank you for the opportunity to review this scoping review protocol, which seems particularly timely as the authors have clearly identified the dearth of synthesised evidence relating to the BFHI and particularly the BFCI. This scoping review using the framework by Arskey and O’Malley (2005) is clearly described and the rationale for choice of review is defended. The Scoping Review protocol as presented is clearly articulated outlining each step of the systematic review process. As reviewers we have the following suggestions which we propose would enhance the review protocol:

1. The inclusion of the reference to Malawi in the abstract is a distraction and recommend its removal.
2. The conclusion to the scoping review needs to be mirrored in the abstract as there seems to be a disconnect between the global perspective and the research plans articulated for Malawi.
3. The last statement of the Study rationale section refers to empirical research, yet the inclusion criteria includes reviews. This needs to be revised.
4. There is no need to use the abbreviations in the ‘Identifying the research question’ section as this had already been previously addressed.
5. The iterative process for this scoping review is to be commended. However, as per point 2 above, the global perspective needs to be better reflected in stage 1.
6. In Stage 2, it is to be commended that a librarian would be engaged in the search strategy as this will ensure that all relevant databases and appropriate search terms will be utilised.
7. The authors refer to the inclusion of ‘all types of review’, however, we believe that inclusion of reviews should be confined to systematic reviews, metasynthesis or any that have been underpinned by a defined framework such as PRISMA.
8. In relation to point iii) of the inclusion criteria, could greater clarity be provided by referring to the support from the 10 steps to interrogate the evidence as support in general is such a broad concept.
9. In relation to point vii) empirical studies, it would be best to revise if the authors are including reviews.
10. In terms of Exclusion criteria no iii), we suggest that conference proceeding also be included here.
11. In Stage 3, we suggest us of the term papers be employed rather than studies to reflect that more that one paper may be published from a particularly study.
12. In Stage 4, it is important to note that identifying gaps in the literature through a scoping study will not necessarily identify research gaps where the research itself is of poor quality since quality assessment does not form part of the scoping study remit. Therefore we suggest this limitation is acknowledged.
13. On the data extraction plan, the final point (no 11) states ‘key findings’ which seems non specific. The inclusion of sub divisions would aid clarity.
14. In Stage 5, use of the Levsque framework is not clearly articulated, for example, is it the intention to extract data according to the framework?
15. In Stage 6, following on from the above point, reference to the ‘…application of the accessibility framework...’ is not clear.
16. In Stage 6, there is reference to realist research which is not sufficiently contextualised or integrated with the scoping review protocol and maybe consider removing.

Thanking you and wishing you every success with this work

 Patricia and Helen

Is the study design appropriate for the research question?

Yes

Is the rationale for, and objectives of, the study clearly described?

Yes

Are sufficient details of the methods provided to allow replication by others?

Partly

Are the datasets clearly presented in a useable and accessible format?

Not applicable

Reviewer Expertise:

Patricia Leahy-Warren areas of research expertise include but not limited to: breastfeeding; perinatal mental health; transition to parenthood and social support. Helen Mulcahy areas of research include but not limited to: breastfeeding support; child health; and parental concern.

We confirm that we have read this submission and believe that we have an appropriate level of expertise to confirm that it is of an acceptable scientific standard.

# Response

**Published:** 2021-04-06

**Authors:** Aisling Walsh

**Affiliations:** Royal College of Surgeons in Ireland, Ireland

Thank you very much for taking the time to review this protocol and for your considered and helpful feedback. 

1. **The inclusion of the reference to Malawi in the abstract is a distraction and recommend its removal.**
**  **The reference to consultation with a group of stakeholders in Malawi has been removed.

 

1. **The conclusion to the scoping review needs to be mirrored in the abstract as there seems to be a disconnect between the global perspective and the research plans articulated for Malawi.**
**  **The abstract has been amended to reflect the aim to fill the gap in the global literature.

 

1. **The last statement of the Study rationale section refers to empirical research, yet the inclusion criteria includes reviews. This needs to be revised.**
  The inclusion of literature reviews are reviews of empirical research. This has now been amended in the inclusion criteria and also on page 7.

** **

1. **There is no need to use the abbreviations in the ‘Identifying the research question’ section as this had already been previously addressed.**
  This has now been amended.

** **

1. **The iterative process for this scoping review is to be commended. However, as per point 2 above, the global perspective needs to be better reflected in stage 1.**
   We have moved the section which describes the global perspective from the study rationale section to Stage 1.  We also have deleted much of the Malawi background context in Stage 1.

 

1. **In Stage 2, it is to be commended that a librarian would be engaged in the search strategy as this will ensure that all relevant databases and appropriate search terms will be utilised.**

** **

1. **The authors refer to the inclusion of ‘all types of review’, however, we believe that inclusion of reviews should be confined to systematic reviews, metasynthesis or any that have been underpinned by a defined framework such as PRISMA.**
  We have amended this to include all types of reviews of empirical research to ensure that we do not miss any empirical research that could be included in other types of reviews. 
** **

** **

1. **In relation to point iii) of the inclusion criteria, could greater clarity be provided by referring to the support from the 10 steps to interrogate the evidence as support in general is such a broad concept.**
  This criterion has been amended to include: focus on experiences of accessing/delivering supports and services through any of the ten steps of the BFHI and/or BFCI. We have also made other minor amendments to the inclusion criteria.

** **

1. **In relation to point vii) empirical studies, it would be best to revise if the authors are including reviews.**

Literature reviews of empirical research been added here.

1. **In terms of Exclusion criteria no iii), we suggest that conference proceeding also be included here.**
  Conference proceedings, trial registrations, study protocols and letters have now been included as exclusion criteria.

** **

1. **In Stage 3, we suggest us of the term papers be employed rather than studies to reflect that more that one paper may be published from a particularly study.**
  The word ‘studies’ has been replaced with ‘article’ where appropriate throughout the protocol.

** **

1. **In Stage 4, it is important to note that identifying gaps in the literature through a scoping study will not necessarily identify research gaps where the research itself is of poor quality since quality assessment does** **not form part of the scoping study remit. Therefore we suggest this limitation is acknowledged.**
  This is now acknowledged in Stage 4.

 

1. **On the data extraction plan, the final point (no 11) states ‘key findings’ which seems non specific. The inclusion of sub divisions would aid clarity.**
  Thank you for this useful point. Following this feedback, we have decided to chart the data according to the PAGER (Patterns, Advances, Gaps, Evidence for practice and Research recommendations) framework (Bradbury-Jones et al., 2021). This framework is a methodological framework which will enable us to analyse and report review findings. This has been detailed in the abstract and in Stage 5.

 

1. ** In Stage 5, use of the Levesque framework is not clearly articulated, for example, is it the intention to extract data according to the framework?**
  Having reflected on the reviewers comments, we have taken out the equity dimension to the review. From an initial scoping of the literature, it is becoming apparent that equity is not sufficiently examined in the literature. We feel that the PAGER framework is a more appropriate overall framework to chart, analyse and report the findings. References to the equity dimension and to the Levesque framework have been removed from the protocol.  

 

1. **In Stage 6, following on from the above point, reference to the ‘…application of the accessibility framework...’ is not clear.**
  We have removed this. In order to highlight the global focus and relevance of the review, we will consult with stakeholders at global and WHO regional levels, including practitioners, researchers and policy makers. ****

** **

1. **In Stage 6, there is reference to realist research which is not sufficiently contextualised or integrated with the scoping review protocol and maybe consider removing.**

We have removed the reference to realist research.
